# Biomedical Applications of Bacterial Exopolysaccharides: A Review

**DOI:** 10.3390/polym13040530

**Published:** 2021-02-10

**Authors:** Masrina Mohd Nadzir, Retno Wahyu Nurhayati, Farhana Nazira Idris, Minh Hong Nguyen

**Affiliations:** 1 School of Chemical Engineering, Engineering Campus, Universiti Sains Malaysia, Nibong Tebal 14300, Malaysia; farhana_nazira@yahoo.com; 2 Department of Chemical Engineering, Faculty of Engineering, Universitas Indonesia, Depok 16424, Indonesia; retno.wahyu01@ui.ac.id; 3 Stem Cell and Tissue Engineering Research Cluster, Indonesian Medical Education and Research Institute, Faculty of Medicine, Universitas Indonesia, Jl. Salemba Raya No. 6, Jakarta 10430, Indonesia; 4 Faculty of Biotechnology, Chemistry and Environmental Engineering, Phenikaa University, Hanoi 12116, Vietnam; minh.nguyenhong@phenikaa-uni.edu.vn; 5 Bioresource Research Center, Phenikaa University, Hanoi 12116, Vietnam

**Keywords:** alginate, bacteria, biomedical, cellulose, dextran, exopolysaccharides, gellan, hyaluronic acid, levan, xanthan gum

## Abstract

Bacterial exopolysaccharides (EPSs) are an essential group of compounds secreted by bacteria. These versatile EPSs are utilized individually or in combination with different materials for a broad range of biomedical field functions. The various applications can be explained by the vast number of derivatives with useful properties that can be controlled. This review offers insight on the current research trend of nine commonly used EPSs, their biosynthesis pathways, their characteristics, and the biomedical applications of these relevant bioproducts.

## 1. Introduction

Bacterial exopolysaccharides (EPSs) are high molecular weight carbohydrate biopolymers often secreted by cells into the extracellular environment. EPSs provide various functions that are useful to bacteria. Some bacterial EPSs with valuable physicochemical properties have already been utilized for biomedical applications. For instance, among all the reported EPSs, bacterial cellulose (BC) has been the most studied as a material for dental implants, wound dressing, and a temporary skin substitute, leading to the commercialization of biomedical products [1]. The global microbial and BC market was valued at US$ 250 million in 2017 and estimated to reach US$ 680 million by the end of 2025 [2]. Another EPS, xanthan gum, is also showing increasing market value, with the global xanthan gum market valued at ~US$ 1 billion in 2019 and expected to reach ~US$ 1.5 billion in 2027 [3].

**Figure 1:**
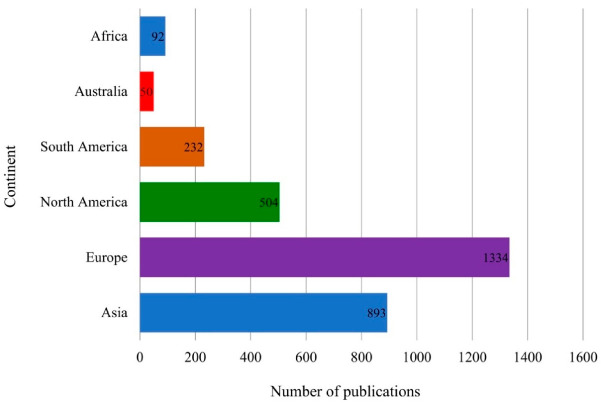
Research trend of microbial exopolysaccharides published in Scopus based on continents.

Bacteria are the most commonly used EPS source because they reproduce rapidly, are easily renewed, and are compatible with most EPS isolation methods. Among the different continents, the most research on EPS, as indexed in Scopus, has been in Europe (1334 publications, 43%), followed by Asia (893 publications, 29%), North America (504 publications, 16%), South America (232 publications, 7%), Africa (92 publications, 3%), and Australia (50 publications, 2%) [4] (Figure 1). The research trend might depend on the bioresource of EPS, advances in industries and technology, the knowledge of EPS, and the political stability of these regions. The interest in EPS has resulted in several EPS-producing bacteria patents, such as *Pediococcus acidilactici* 05b0111 [5], *Pseudomonas* sp. CECT8437 [6], *Shigella sonnei* [7], and *Lactobacillus rhamnosus* [8].

**Figure 2:**
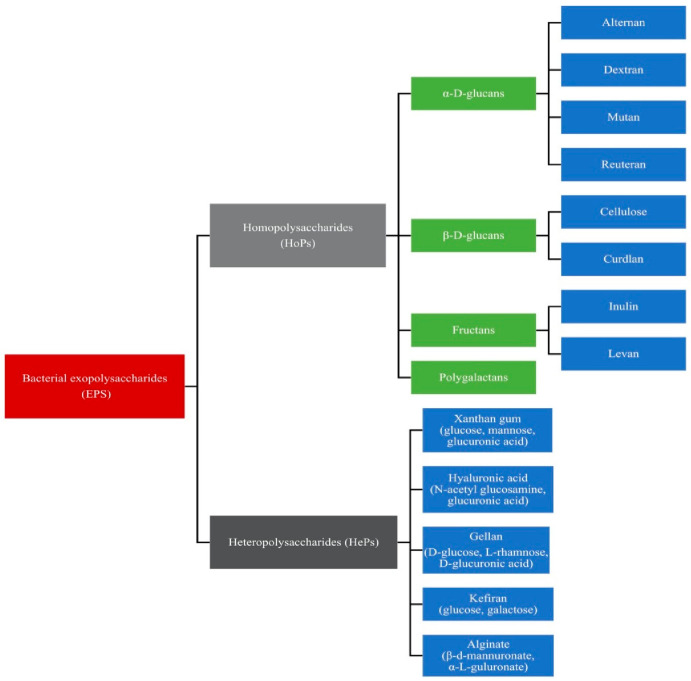
Classification of bacterial exopolysaccharides.

Bacterial EPSs are loosely-attached slime layers that can be removed easily from cells. The strains, the composition of the medium, and the conditions of the culture, such as temperature, pH, and the carbon/nitrogen ratio, determine the amount of EPS produced by bacteria. For example, different monosaccharide compositions, linkages, charges, the presence of repeated side-chains, and substitutions in probiotic bacteria result in various types of EPSs. Generally, EPSs can be grouped into homopolysaccharides (HoPs) and heteropolysaccharides (HePs) [9,10]. Homopolysaccharides are either unbranched or branched and composed of either fructose or glucose. They are categorized into α-D-glucans (e.g., alternan, dextran, and reuteran), β-D-glucans, fructans (e.g., inulin and levan), and polygalactans. Heteropolysaccharides are polymers such as hyaluronic acid (HA), xanthan gum, alginate (ALG), kefiran, and gellan, which consist of more than one type of monosaccharide [9,11]. The classification of bacterial EPSs is shown in Figure 2.

Exopolysaccharides produced by bacteria can promote intestinal health, altering microbes’ composition, enhancing immune activity, and improving blood flow [4,11,12]. Cellulose, dextran, xanthan gum, HA, ALG, kefiran, gellan, levan, and curdlan are some of the microbial EPSs with biomedical applications. The EPS-producing bacteria are *Acetobacter*, *Agrobacterium*, *Bacillus*, *Brenneria*, *Geobacillus*, *Gluconacetobacter*, *Halomonas*, *Lactobacillus*, *Rhizobium*, *Saccharomyces*, *Sarcina*, *Streptococcus*, *Xanthomonas*, and *Zymomonas* [11,12]. Probiotic bacteria (e.g., *Lactobacillus*, *Leuconostoc*, *Lactococcus*, *Bifidobacterium*, *Streptococcus*, and *Enterococcus*) have been used predominantly to synthesis EPSs for various applications. This is because they are considered safe and able to survive among gastric juices, bile, and low pH and colonize in the gastrointestinal tract’s epithelial layer [9,10]. Since the demand for EPSs is increasing due to qualities such as their biocompatibility, biodegradability, and non-toxicity, new EPSs are being formed by blending them with other natural and synthetic polymers, thus motivating researchers to discover novel applications in various areas for future use. Table 1 lists some of the studies conducted on EPSs in the biomedical field. This review discusses the microbial biosynthetic pathways, nine types of commonly used EPSs produced by bacteria, and their applications in biomedical industries, especially for scaffolds, coatings, sealants, and drug delivery systems.

## 2. Microbial Biosynthesis of Exopolysaccharides

**Figure 3:**
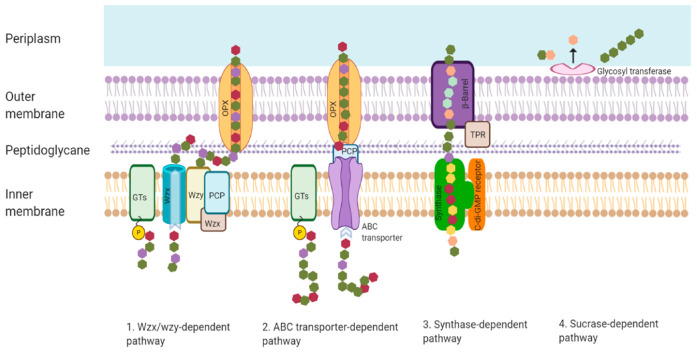
Microbial pathways for EPS synthesis. GTs: glycosyltransferases; PCP: polysaccharide co-polymerase; OPX: outer membrane polysaccharide export; TPR: tetratricopeptide repeat protein.

Biosynthesis of EPSs in bacteria occurs in intra- and extracellular ways. There are four general mechanisms for EPS biosynthesis in bacterial cells, which are the Wzx/Wzy-dependent pathway, the ABC transporter-dependent pathway, the synthase-dependent pathway, and extracellular biosynthesis by sucrase protein [9,16,49,50,51]. The aforementioned microbial pathways are shown in Figure 3. HoPs are generally synthesized by the synthase-based pathway and the extracellular production pathway. In contrast, HePs are generated by the Wzx/Wzy-dependent pathway and the ABC transporter-dependent pathway [9].

### 2.1. Wzx/Wzy-Dependent Pathway

The Wzx/Wzy-dependent pathway consists of three stages: (i) synthesis of nucleotide sugars, (ii) assembly of repeat units, and (iii) polymerization and export [50]. In the first stage, the sugar residues are actively transported into cells and transformed into various monomeric units, which are transferred toward and linked to an undecaprenyl phosphate (Und-P) anchor (C55 lipid carrier) at the inner membrane [52,53]. In the second stage, glycosyltransferases (GTs) link more sugar units to produce repeating units, which translocate across the cytoplasmic membrane by Wzx flippase [16,53,54]. For the last stage, the translocated oligosaccharide units undergo various enzyme modifications, such as methylation and acetylation, and are polymerized to polysaccharides by the Wzy protein [54]. The polysaccharides assembled via the Wzx/Wzy-dependent pathway are HePs containing highly diverse sugar units [55,56]. The assembled polysaccharides are released to the cell surface by ABC transporters [57]. In probiotic bacteria, the HePs are gellan, xanthan, and kefiran, produced by the Wzx/Wzy-dependent pathway [9].

### 2.2. ABC Transporter-Dependent Pathway

In the ABC transporter-dependent pathway, such as that employed in the synthesis of *Myxococcus xanthus* EPS, the active sugar units are transported to an Und-P molecule at the inner membrane to form an Und-PP-sugar molecule [58]. The full-length polysaccharides are synthesized by specific GTs located at the cytoplasmic side’s inner membrane and then translocated across the inner membrane by a tripartite efflux pump complex in the inner membrane [59]. This pathway is mainly involved in synthesizing capsular polysaccharides [58,60].

### 2.3. Synthase-Dependent Pathway

The polymer products based on the synthase-dependent pathway are HoPs made from a single type of sugar unit, such as bacterial ALG and cellulose [61,62,63]. In the synthase-dependent pathway, the assembly of UDP-glucose units occurs by a membrane-embedded synthase/inner membrane transporter bacterial cellulose synthesis (bcs) A [61]. Bacterial cellulose synthesis operons are highly variable and species-dependent [50].

### 2.4. Extracellular Biosynthesis by Sucrase Protein

In the extracellular biosynthesis pathway, sucrose is transformed by extracellular sucrase enzymes into monomeric units outside the cellular outer membrane [62]. The GTs polymerize the monosaccharide units into glucan (dextran) and fructan (levan) with different branches [64]. In probiotic bacterial cells, glucan sucrases are categorized as alternan sucrases (alternan), dextran sucrases (dextran), mutan sucrases (mutan), and reuteran sucrases (reuteran). In contrast, fructan sucrases are separated into levan sucrases (levan) and inulin sucrases (inulin) [65]. As a result, the EPSs synthesized in probiotic bacteria by the extracellular biosynthesis pathway are HoPs. For example, the glucose monomer is the component of dextran, mutan, alternan, reuteran, and curdlan, while levan and polygalactans are made from fructose and galactose, respectively [9]. The synthesized EPSs are released to the extracellular environment [56].

## 3. Types and Properties of Bacterial Exopolysaccharides

Commonly used EPSs in the biomedical field include cellulose, dextran, xanthan gum, HA, ALG, kefiran, gellan, levan, and curdlan. These EPSs have various structural and physicochemical properties that can be tailored to fit multiple applications. Furthermore, most of these EPSs have an established fermentation process, which is critical to fulfilling the increasing global demand.

### 3.1. Cellulose

BC is a fibrous material consisting of a three-dimensional non-woven network of nanofibrils. This EPS is made up of (1→4)-D-anhydroglucopyranose chains bounded through ß-glycosidic linkages. Cellulose type I of BC consists of parallel chains formed by a network of intra- and intermolecular hydrogen-bonding, van der Waals interactions, and hydrophobic interactions [66]. Cellulose type II can be obtained from BC through a treatment with sodium hydroxide (5–30 wt%) to form anti-parallel packing. Cellulose type II’s stability is mainly due to hydrogen bond packing [67,68]. In contrast to plant cellulose, BC does not contain impurities (e.g., lignin, pectin, and hemicellulose); thus, additional purification is unnecessary prior to using the cellulose for practical applications. Furthermore, BC’s structural, physicochemical, and mechanical characteristics are better than those of plant cellulose [69]. BC’s excellent mechanical features can be attributed to the high surface area of the BC fiber due to its small diameter, which is in the range of 20–100 nm [70]. The stress–strain behavior of BC, which resembles that of soft tissue [71,72], its high liquid loading capacity, and its biocompatibility are some of the reasons that BC is widely used in biomedical industries.

Bacterial cellulose is produced extracellularly by Gram-negative bacteria of the genera *Acetobacter*, *Achromobacter*, *Aerobacter*, *Agrobacterium*, *Alcaligenes*, *Azobacter*, *Gluconacetobacter*, *Pseudomonas*, *Rhizobium*, *Salmonella*, and *Sarcina* [1]. Bacterial cellulose production is affected by the culture method, the microbial strain, pH, temperature, and the carbon source [73]. Interestingly, the carbon source can influence the water holding capacity, mechanical properties, and the molecular weight of BC while not affecting its chemical structure [74,75,76,77,78,79].

### 3.2. Dextran

Dextran is a type of HoP composed of main chains with α-(1,6) linkages and α-(1,2), α-(1,3), and α-(1,4) branch linkages [80]. The size of commercial dextran ranges from 5 to 500 kDa. This biocompatible EPS with anticancer, antibacterial, and antifungal properties has a broad size range, contributing to its application in numerous industries, such as the food and biomedical industries [18,81,82]. Dextran is soluble in ethylene glycol, formamide, glycerol, methyl sulfoxide, and water [17]. The morphology of freeze-dried dextran can be either porous or non-porous, depending on the microbial strain [83]. The morphology of dextran influences its ability to hold water and form a gel. Furthermore, dextran exhibits liquid-like behavior at a low concentration (2.5% (*w*/*v*)), and at a high concentration (>5% (*w*/*v*)), it exhibits both gel-and liquid-like behaviors [84].

Dextran is synthesized by lactic acid bacteria (LAB) belonging to the *Weisella*, *Lactobacillus*, *Pediococcus*, and *Leuconostoc* genera in a sucrose-rich media [83,85,86]. The molecular weight and the yield of dextran production depend on the process parameters, namely, the carbon concentration, temperature, and the microbial strain [87].

### 3.3. Xanthan Gum

Xanthan gum is composed of a ß-(1→4)-D-glucopyranose glucan backbone with (1→3)-α-D-mannopyranose-(2→1)-ß-D-glucoronic acid-(4→1)-ß-D-mannopyranose side chains on alternating residues. This non-linear anionic EPS has a molecular weight in the range of 2 × 10^2^ to 20 × 10^6^ Da. It comprises repeats of a five-monosaccharide unit of two D-glucose, two D-mannose, and one D-glucuronic acid [88,89]. Xanthan is water-soluble, and its solutions exhibit high pseudoplastic flow even at low concentrations [90].

This EPS is generated by Gram-negative bacteria belonging to the genus *Xanthomonas* using aerobic fermentation [91]. Like the other EPS, xanthan gum’s yield and quality can be modified using different bacterial strains and a fermentation environment (e.g., carbon source, cell immobilization, temperature, pH, mixing speed, inoculum volume, and airflow rate). The carbon source is an essential factor in microbial xanthan fermentation, which acts as an energy source and is used in HeP synthesis, the secondary metabolites of the microorganism [91,92]. The immobilization of bacteria cells (e.g., *Xanthomonas campestris* and *Xanthomonas pelargonii*) on calcium alginate-based beads showed higher xanthan titers compared to free cells, irrespective of the carbon source, due to greater culture medium access and oxygen mass transfer [93].

### 3.4. Hyaluronic Acid

Hyaluronic acid is composed of disaccharide subunits of β-D (1→4) *N*-acetyl-β-d-glucosamine units and β-D (1→3) glucuronic acid [94]. This EPS has a molecular weight ranging from 10^4^ to 10^7^ Da, depending on its sources [95]. This anionic EPS is highly biocompatible, non-immunogenic, and highly moisture-absorptive; has a viscoelastic nature; and does not produce harmful products when degraded [96]. High molecular weight (>10 kDa) HA has good moisture retention, viscoelasticity, and mucoadhesion. On the other hand, HA with a fairly low molecular weight (2–3.5 kDa) has been revealed to promote angiogenesis, induce inflammatory mediator expression, and inhibit tumor growth [97]. Hyaluronic acid is also water-soluble and forms a highly non-Newtonian solution that behaves in a gel-like manner [98,99].

Hyaluronic acid is produced by bacterial pathogens like *Pasteurella multocida* and Gram-positive *Streptococcus* Groups A and C [100,101]. Optimal bacterial growth occurred at a temperature of 37 °C and neutral pH. However, the best HA productivity and molecular weight were observed in suboptimal growth conditions, when the growth inhibition was not connected to lower carbon uptake [98]. The carbon source can be sucrose or glucose, with the latter being the preferred source [101]. The molecular weight of HA can be enhanced by a combination of mild shear stress culture conditions and a high dissolved oxygen level [95].

### 3.5. Alginate

Alginate is made up of the uronic acid stereoisomers α-l-guluronic acid (G) and β-d-mannuronic acid (M) [102]. This EPS possesses a significant degree of physicochemical assortment, which influences its characteristics and determines its prospective applicability. Alginate is available in numerous compositions, molecular weights, and distribution forms of M- and G-blocks. Viscosity, sol–gel transition, and water-uptake ability determine their physicochemical properties. On average, commercial ALG’s molecular weight varies between 33,000 and 400,000 g/mol. The ALG extracted from diverse sources varies in M and G residues as well as the length of each block. Raising the ALG G-block content or molecular weight will generally lead to more robust and brittle ALG gels [103,104]. A heat-stable irreversible gel can be formed when chelating ALG with metal ions such as Ca^2+^ [105].

Alginate can be obtained from bacterial species of the genera *Azobacter* and *Pseudomonas* [106,107]. ALG’s molecular weight is strongly influenced by the stirring speed and the culture’s dissolved oxygen tension. It was reported that *Azobacter vinelandii* produced high molecular weight ALG (680,000 g/g mol) at a low agitation speed of 300 rev./min, whereas low molecular weight ALG (352,000 g/g mol) was produced at a high agitation speed (700 rev./min) [108]. Typical carbon sources for the biosynthesis of ALG are glucose or sucrose, with sucrose being the preferred carbon source [29].

### 3.6. Kefiran

This branched EPS is composed of D-galactose (Gal) and D-glucose (Glc) in almost a 1:1 M ratio [109]. The backbone of kefiran is constituted by (1→6)-linked Glc, (1→3)-linked Gal, (1→4)-linked Gal, (1→4)-linked Glc, and (1→2, 6)-linked Gal. The Glc branches are attached to the O-2 of Gal residues. The chain backbone is terminated by a Glc residue [110,111]. Solid-state kefiran is a semi-crystalline polymer with an approximate degree of crystallinity of ca. 30% [109,112]. The molecular weight of kefiran is in the range of 50–15,000 kDa and depends on the carbon source, the conditions of isolation, and purification [113,114]. This water-soluble EPS is reasonably resistant to hydrolysis. Furthermore, kefiran gel can form in aqueous solutions containing ethanol [115]. Kefiran has been reported to have antibacterial capability and antioxidant properties, to support cell metabolic activity, and to assist in cell proliferation, indicating its suitability as a biomaterial for biomedical applications [116,117].

This EPS can be found in the pure culture of *Lactobacillus kefiranofaciens* (*L. kefiranofaciens*) or in kefir grains under aerobic environments and in the mixed cultures of *Saccharomyces cerevisiae* with *L. kefiranofaciens* under anaerobic environments [118,119,120,121]. Compared to other polysaccharides of microbial origin, kefiran’s main advantage is that it is produced from LAB, generally recognized as safe [122,123]. The carbon source, pH, and temperature are critical for the production of kefiran. It has been shown that lactose is the best carbon source for kefiran production [124,125]. The highest output of kefiran has been acquired in the temperature range of 20–30 °C and pH values between 5 and 6 [118,126,127,128].

### 3.7. Gellan

Gellan or gellan gum is an anionic, linear EPS with repeating units of a tetrasaccharide of D-glucose, D-glucuronic acid, D-glucose, and L-rhamnose [129,130,131,132,133,134]. This EPS is soluble in water, in which it forms a viscous solution [135]. Gellan has an average molecular weight of about 500 kDa [129]. The native form of gellan contains two acyl substituents. Alkaline hydrolysis can remove these acyl substituents, forming deacetylated gellan [130,136]. The high-acetyl gellan and the partially deacetylated form provide soft, elastic, and thermo-reversible gels upon cooling from 65 °C. On the other hand, the low-acetyl gellan (highly deacetylated) forms rigid and brittle gels upon cooling to below 40 °C [137,138,139]. Gellan forms a physical gel due to a shift from a random coil to double-helix upon cooling. Strong gels are developed if the cation is present during the sol–gel transformation [140].

Gellan can be obtained from the *Sphingomonas* and *Pseudomonas* genera [141,142,143,144]. The carbon source, nitrogen concentration, and the culture medium’s pH perform a critical role in the biosynthesis of gellan. The carbon source (e.g., glucose, fructose, mannitol, and sucrose) can be utilized by itself or in combination. Usually, the quantity of the carbon source varies between 2 and 4% by mass [145]. The best gellan production was obtained when the bacteria were supplied with minimal nitrogen and plentiful carbon [146]. Cell growth and product formation were substantially affected by the pH. It was recommended that the pH used for gellan production be near or at neutral pH [147]. An environment that is highly acidic or highly alkaline lowers the cell growth and ultimately the production of gellan [147,148,149].

### 3.8. Levan

Levan consists of fructose units connected by β-2,6-glycoside bonds in the backbone and β-2,1 in its branches. This amphiphilic polymer is extremely soluble in water, and its solubility can be improved by increasing the water’s temperature [150]. Levan is insoluble in the majority of organic solvents, apart from dimethyl sulfoxide (DMSO) [151]. The high solubility of bacterial levan with a high molecular weight in water is attributable to the polymer’s highly branched shape [152]. Although levan is heat stable and has a melting point temperature of 225 °C [153], pure levan film has inferior mechanical properties. The high molecular weight of levan, accompanied by its highly branched and compact globular shape, does not allow substantial intermolecular entanglement, which leads to a brittle levan-based material with a low tensile modulus [154,155]. In the biomedical sectors, levan acquires countless applications due to its antibacterial properties, biocompatibility, and antioxidant activity [156,157].

The bacteria genera that produce levan include *Bacillus, Erwinia, Pseudomonas*, and *Zymomonas* [156,158,159,160,161,162,163,164]. Microbial levan is made from a sucrose-based substrate by the action of one of the microorganism’s secreted enzyme, levansucrase (EC2.4.1.10) [165]. Levan’s molecular weight varies with the type of bacteria used for its synthesis and the cultivation parameters. Levan produced by the *Bacillus aryabhattai* GYC2-3 strain had a high average molecular weight (5.317 × 10^7^ Da) [166], while levan from *Bacillus licheniformis* 8-37-0-1 had a low molecular weight (2.826 × 10^4^ Da) [160]. The sucrose concentration was found to be the critical component in modulating the molecular weight of synthesized levan. Using a low sucrose concentration (20 g/L) in a culture of *Bacillus subtilis* (natto) Takahashi resulted in predominantly high molecular weight levan (>2 × 10^6^ Da). In contrast, low molecular weight levan (6–9 × 10^3^ Da) was the prevalent EPS when a high sucrose concentration (400 g/L) was used [167].

### 3.9. Curdlan

Curdlan is a linear (triple-helix) polysaccharide comprising 1,3-β-linked D-glucose units [168]. Curdlan powder is insoluble in cold water due to the existence of vast intra-/intermolecular hydrogen bonds. However, it easily dissolves in DMSO and alkaline solutions. Curdlan powder is dispersible in hot water and forms a thermo-reversible or thermo-irreversible gel, subject to the heating temperature. A thermo-reversible gel is formed when the curdlan dispersion is heated to 55–60 °C and then cooled below 40 °C. This EPS will form a thermo-irreversible gel upon heating in an aqueous suspension at elevated temperatures (80 °C or above) followed by cooling. The gelation of curdlan has been attributed to hydrogen bonding and possible hydrophobic interactions [169,170]. Furthermore, it was revealed that curdlan structures changed with the heating temperature. Curdlan triple-stranded helixes primarily underwent hydration and swelling at 40 °C and were segregated from each other at 50 °C. These triple-stranded helixes dissociated into partially opened triple-helical chains and single-helical chains at 60 and 70 °C. At 80 and 90 °C, large proportions of the dissociated single-helical chain were present [171]. This unique ability of curdlan is rarely seen for many other polysaccharides, which is why it is extensively applied in pharmaceutical industries.

Curdlan is produced by *Alcaligenes faecalis*, *Rhizobium radiobacter*, and *Agrobacterium* species in a nitrogen-limiting environment with glucose or sucrose as the carbon source [172,173,174,175]. Factors known to affect curdlan biosynthesis include the nitrogen source, the carbon source, oxygen supply, and pH [176,177,178,179]. The carbon source also influences curdlan’s molecular weight and gel strength. However, it does not affect curdlan’s structural and thermal properties. A culture of *Agrobacterium* sp. DH-2 in a xylose medium resulted in curdlan with a molecular weight of 1.59 × 10^6^ Da and a gel strength of 989.2 g/cm^2^, while a culture in a sucrose medium resulted in curdlan with a weight of 1.10 × 10^6^ Da and gel strength of 672.8 g/cm. The curdlan gel strength correlates positively with its molecular weight [180].

## 4. Biomedical Applications

The biocompatibility and functional properties of EPSs are important factors that promote their use in various biomedical applications, such as scaffolds, drug delivery systems, coating materials for medical devices, and surgical sealants. An EPS can be used in its native structure, cross-linked, or tailored with various bioactive materials.

### 4.1. Bacterial Exopolysaccharides as a Scaffold

The success of tissue engineering for regenerative medicine applications is determined by the right combination of stem cells, growth factors, and scaffolds. As natural-based materials, EPSs have shown good biocompatibility, biodegradability, and mechanical strength, which are beneficial for the formation of biological scaffolds. Gellan hydrogels tailored with hydroxyapatite have been developed for bone tissue engineering [181]. A low-acyl gellan solution was heated and mixed with hydroxyapatite. After cooling down at room temperature, the gel was formed, and then it was freeze-dried to create a spongy construct. In vitro analysis showed that human adipose stem cells seeded in the scaffold were alive after 21 days of culturing.

**Figure 4:**
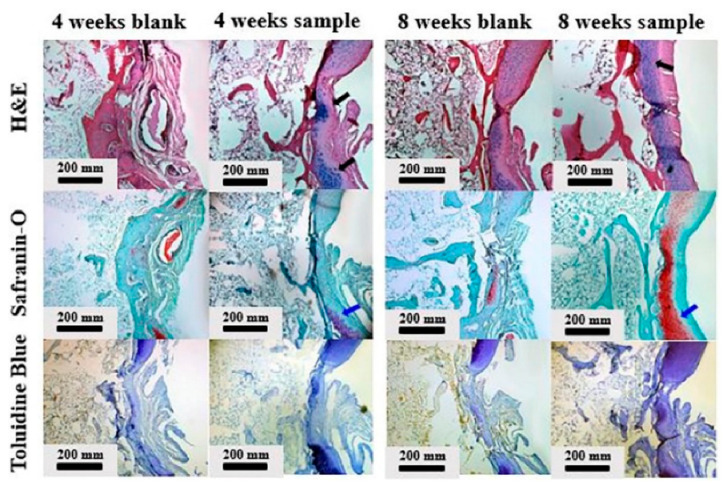
Histological images of an in vivo model at 4 weeks and 8 weeks (scale bar = 200 mm). The black arrows indicate well-formed new tissue. The blue arrows indicate red Safranin-O staining, which dyes an acidic proteoglycan. Reprinted from Kim et al. [38]. Copyright (2019), with permission from Elsevier.

Kim et al. [38] developed gellan hydrogel to encapsulate chondrocyte for cartilage regeneration. Hyaluronic acid was added to complement gellan’s limited biological ability to facilitate chondrocyte growth and attachment. The mechanical testing of composite hydrogels showed that increasing the HA content reduced the compressive strength and accelerated degradation. Accordingly, the ratio of gellan/HA should be controlled to obtain optimal cartilage formation. Gellan/HA composite hydrogels were shown to promote cartilage regeneration in rabbit models. Figure 4 shows that the blank group displayed unevenly formed tissue 4 weeks after implantation, and the regenerated tissue was not well attached to the original cartilage. On the other hand, the hydrogel-implanted group showed well-formed new tissue and showed canonical pericellular matrices around the chondrocytes.

A gellan/HA composite scaffold was developed for skin regeneration [27]. The scaffold was prepared by mixing, molding, freezing, and drying to fabricate a spongy network structure of gellan/HA. Human adipose stem cells and microvascular endothelial cells were seeded to evaluate the gellan/HA scaffold’s ability to facilitate skin regeneration. Implantation of the scaffold in full-thickness excisional wounded mice resulted in the formation of dense granulation tissue. Furthermore, the scaffold significantly promoted re-epithelialization and vascular formation in the transplanted group relative to the untreated group.

Alginate is a well-known hydrogel material extracted from bacterial or brown algae cell walls. Alginate–chitosan hydrogels’ feasibility for human stem cell encapsulation has been reported [30]. A study reported ALG-based hydrogel’s potency for inducing retinal pigment epithelium (RPE) regeneration [31]. The scaffold was loaded with taurine, a neurotransmitter in retina tissue, to promote RPE cell immune protection. The taurine-loaded ALG hydrogel showed a promising effect on RPE cell migration and proliferation in in vitro analyses. Moreover, the scaffold exhibited good biocompatibility and biodegradability when implanted in nude mice.

Radhouani et al. [33] fabricated a kefiran-based scaffold with a freeze-drying technique. The bacterial kefiran was molded to form a stable, elastic, and porous 3D construct. Their study demonstrated that the kefiran-based scaffold was biocompatible with human adipose-derived stem cells and could be used for controlled drug release of diclofenac.

Avsar et al. [40] employed an electrospinning technique to develop fibrous levan-polycaprolactone and levan–polyethyleneoxide matrices. The addition of bacterial levan improved the elongation at break and elastic modulus of polycaprolactone and polyethyleneoxide fibers. In vitro assessment showed the cytocompatibility of levan-based matrices with mouse fibroblast L929 and human umbilical vein endothelial cell lines. The study proposed the future use of levan-based scaffolds for cardiac tissue engineering.

### 4.2. Drug Delivery System

**Figure 5:**
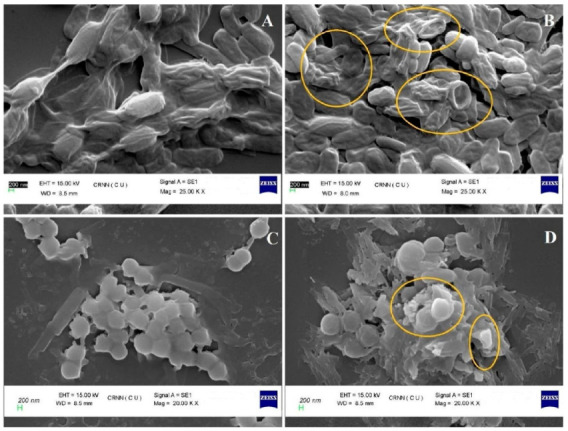
Scanning electron microscopy (SEM) images of (**A**) dead and shriveled *Escherichia coli* treated with ciprofloxacin alone; (**B**) more affected cells (marked) by ciprofloxacin with surfactant EPS; (**C**) dead *Staphylococcus aureus* with disruption of coccoid morphology by ciprofloxacin alone; (**D**) more affected cells (marked) by ciprofloxacin with surfactant EPS. Reprinted from Sengupta et al. [14]. Copyright (2020), with permission from Elsevier.

Exopolysaccharides are prospective candidates for drug delivery systems due to their bioactive function and capacity as a drug carrier. They can serve as biosurfactants, with potential use in cosmetics products for functions such as oil control, and antibacterial agents [182]. The EPS extracted from *Ochrobactrum pseudintermedium* C1 was reported to inhibit pathogenic bacterial growth, as shown in Figure 5 [14]. The antibacterial activity was enhanced when combined with a standard antibiotic, e.g., ciprofloxacin, suggesting its potential use as an adjuvant to prevent antibiotic resistance. Gold nanoparticles functionalized with bacterial EPS exhibited more potent bactericidal activity than EPS alone [183].

A bacterial EPS, curdlan, was reported to have the ability to inhibit *Mycobacterium tuberculosis* (Mtb) growth based on in vitro and in vivo studies [46]. Curdlan administration was able to activate macrophages in Mtb-infected mice via nitric oxide production. In a separate study, Inturri et al. [184] reported that EPS derived from *Bifidobacterium longum* W11 stimulated cytokine production from peripheral blood mononuclear cells. Exopolysaccharides derived from *Lactobacillus* sp. exhibited potent immunomodulatory and antioxidant activities [95]. The antioxidant activities of *Lactobacillus* EPS were demonstrated through the chelation of ferrous ions, inhibition of lipid peroxidation, radical scavenging activity, and reducing capacity.

Besides serving as a bioactive agent, bacterial EPS is a potential carrier for valuable medicine, including growth factors and antitumor drugs. Although its function as a vehicle is similar, the fabrication of EPS as a drug carrier is simpler than its fabrication for biological scaffolds loaded with viable cells. As drug carriers, EPSs can be modified to facilitate controlled drug release, increase drug shelf-life in the body, and improve drug efficacy.

Antibiotics are widely used as a model for drug delivery release with bacterial EPSs. Kefiran–ALG microspheres were developed to facilitate the controlled release of a broad-spectrum antibiotic, ciprofloxacin [128]. This study reported that kefiran–ALG encapsulation was able to protect ciprofloxacin from gastric conditions based on in vitro experiments. In another report, succinic anhydride-modified xanthan gel was developed to facilitate the sustained release of gentamicin [21]. In vitro analysis showed that the hydrogel was able to maintain the sustained release of gentamicin for 9 days under physiological conditions. In addition to maintaining antibiotic efficacy, the gentamicin-loaded gel showed cytocompatibility based on in vitro analysis with human lens epithelial cell culture and an in vivo study with subcutaneously implanted rabbit models.

Bacterial cellulose is a promising substitute for plant-based material in dental medical applications due to its high tensile strength, absorption, and biocompatibility [185]. A recent report from Inoue et al. [186] showed the potency of bacterial cellulose for the prolonged release of chlorhexidine, an antibacterial drug. The inclusion complex of chlorhexidine with β-cyclodextrin was created and then incorporated into a cellulose membrane. The strong chemical interactions between the complex and the cellulose structure successfully increased the drug release by up to 10-fold compared to unmodified cellulose.

**Figure 6:**
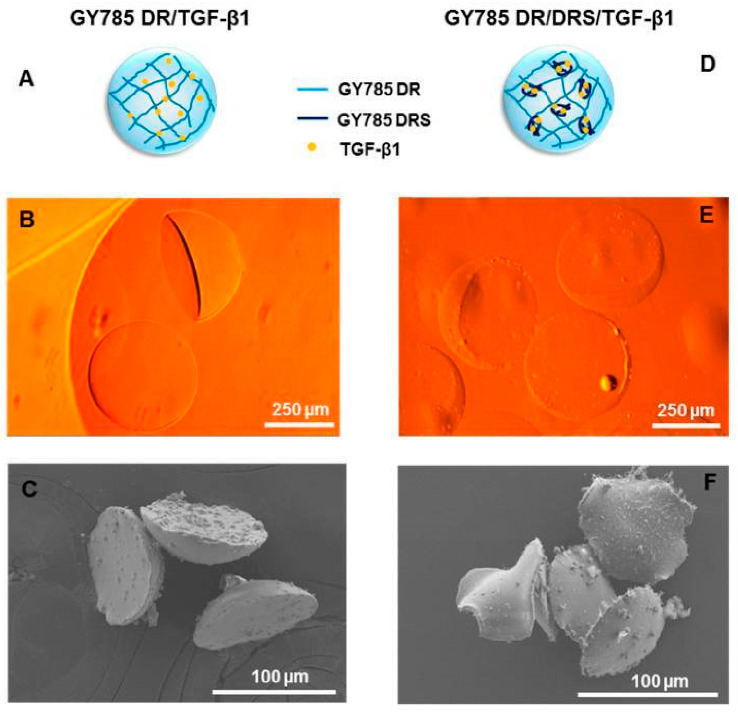
Single GY785 DR/TGF-β1 microcarrier: (**A**) schematic representation of the microparticle section, (**B**) phase-contrast optical image, and (**C**) SEM image. Double GY785 DR/DRS/TGF-β1 microcarrier: (**D**) schematic representation of the microparticle section, (**E**) phase-contrast optical image, and (**F**) SEM image. Figure and caption reused from Zykwinska et al. [187]. Used under the Creative Commons License (http://creativecommons.org/licenses/by/4.0/ (accessed on 28 January 2021)).

Zykwinka et al. [187] developed EPS-based microparticles with a microfluidic approach (Figure 6). The EPS, namely, GY785, was extracted from a hydrothermal bacterium, *Alteromonas infernusa*. The EPS microparticles were loaded with TGF-β1, a valuable growth factor for cartilage regeneration. As analyzed in in vitro studies, this strategy successfully improved the bioactivity and bioavailability of TGF-β1.

The development of nanoparticles of cholesterol-conjugated carboxymethyl curdlan for use as a drug carrier was reported by Li et al. [45]. The curdlan-based nanoparticles were used to entrap an antitumor drug and epirubicin. Their study showed that EPS nanoparticles could prolong drug retention in the body and subsequently improve epirubicin’s antitumor capacity, based on in vivo experiments with Wistar rats.

Qiu et al. [13] constructed a wound healing membrane from bacterial cellulose loaded with vaccarin, an angiogenesis-promoting drug. The vaccarin-loaded cellulose membrane showed improved physical and mechanical properties compared to the cellulose membrane. In vitro analysis with mouse fibroblast cells, L929, showed that both vaccarin-loaded and native cellulose membranes did not exhibit any toxicity effects. The vaccarin-loaded cellulose membrane had a better healing effect than the unloaded membrane, as observed in animal studies with ICR male mice. Histological analysis indicated that vaccarin promoted neovascularization and epithelization in the skin-wounded mice models.

### 4.3. Coating Materials for Medical Devices

Medical devices, including biosensors and prosthetic implants, have significantly improved with modern medical technologies. Typically, medical devices are manufactured from mechanically stable polymers or metal to improve their life expectancy and prevent repetitive surgery. However, the stiffness of durable materials can promote immune response that can be fatal. Exopolysaccharides, primarily dextran, have been used as a coating material for improving the biocompatibility of medical devices.

**Figure 7:**
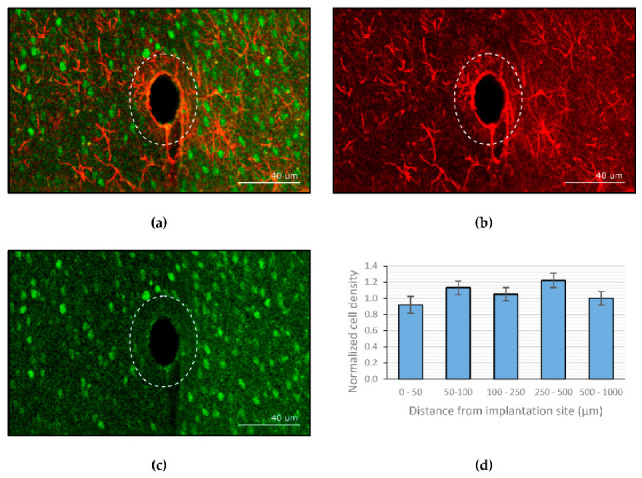
Confocal imaging of brain slices: (**a**) overlay of both the glial fibrillary acidic protein (GFAP) and neuronal nuclei (NeuN) stained channels. (**b**) GFAP channel. (**c**) NeuN channel. (**d**) Normalized neuronal density relative to the site of implantation (the error bars represent the standard deviation, *n* = 6). Figure and caption reused from Kil et al. [188]. Used under the Creative Commons License (http://creativecommons.org/licenses/by/4.0/ (accessed on 28 January 2021)).

Kil et al. [188] explored the possibility of using dextran as a coating material for neural probes. The molecular weight and coating thickness of dextran can be adjusted to control the stiffness and degradation time. This study reported that scar tissue was rarely formed in Wistar rats after 4 months of implantation with a dextran-coated neural probe. Figure 7 shows the average neuronal cell density at several distances from the site of implantation, normalized to the density in the outer concentric circle. The counting of viable neurons in the vicinity of the implant showed that there was no significant decline in neuron density when approaching the implant.

Dextran coating can be loaded with bioactive materials, including growth factors. Noel et al. [189] immobilized VEGF in dextran coating for vascular application. The deposition of VEGF in a vascular graft could selectively attract endothelial cells to adhere to vascular implants, therefore potentially promoting vascular regeneration.

The corrosion behavior of metal remains challenging, although it has been widely used for bone implants. A study by Saveleva et al. [190] reported that coating a titanium-based implant with organic polymers, e.g., dextran, could improve corrosion resistance against simulated body fluid.

### 4.4. Surgical Sealant

**Figure 8:**
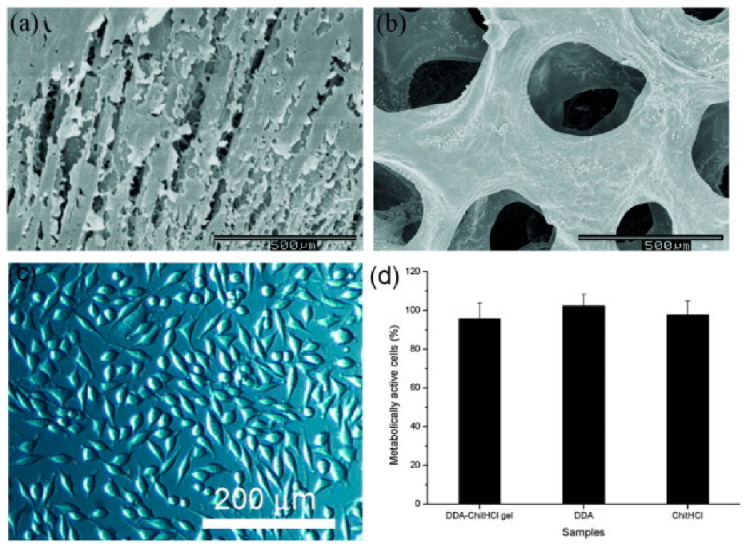
Scanning electron microscopy images showing (**a**) surface morphology and (**b**) internal structure of the representative lyophilized gel prepared using ChitHCl (5 wt%) and DDA50 (10 wt%); (**c**) optical microscope images of L929 mouse fibroblast cells exposed to gels (**d**) and variation in the percentage of metabolically active cells upon exposure to the gel extract and its components. Reprinted from Balakrishnan et al. [20]. Copyright (2017), with permission from Elsevier.

The surgical sealant is an advanced technology to stop bleeding during an invasive procedure. Adhesive biomaterials such as fibrin, chitosan, and dextran are potential materials for surgical sealants. Balakrishnan et al. [20] explained that dextran–chitosan in situ gelling provided good adhesive properties with low cytotoxicity and minimal swelling. Figure 8 shows the structure of gel prepared from 5 wt% ChitHCl and 10 wt% DDA50, in which the fibroblast cells of L_929_ mice maintained their normal spindle shape, proving the cytocompatibility of the gels. For cytotoxicity, after 24 h of contact with the material extract, 95.8 ± 8.06% of cells were metabolically active compared to cells without the material extract. ChitHCl (5 wt%) and DDA (10 wt%) demonstrated 97.6 ± 7.12 and 102.3 ± 5.9% metabolically active cells. This study was evaluated in liver-injured rabbits, and it showed that dextran–chitosan composite could serve as a great adhesive glue and possessed low cytotoxicity.

Among bacterial EPSs, sulfated levan shows promise for future use in cardiac tissue engineering. This is because of its excellent biocompatibility and anticoagulant activity [42]. A study developed adhesive free-standing multilayer films from sulfated levan combined with ALG and chitosan [191]. The presence of sulfated levan significantly improved the mechanical strength and adhesiveness of the constructed adhesive films. The multilayer films were cytocompatible and myoconductive, as evaluated through in vitro testing with a myoblast cell line, C1C12. These results suggested the potency of the sulfated levan–ALG–chitosan membrane for cardiac tissue application.

## 5. Challenges and Future Perspective

Most of the EPS findings lack precise structure–function relationships for biological functions; thus, it is hard to commercialize new EPSs [10]. Detailed information on the EPS structure and bacterial strain is needed since different bacterial strains produce different EPS structures and thus different biological effects.

The pathogenicity of some of the bacteria that produce EPSs is also one of the concerns that hinder the large-scale production and commercialization of certain EPSs. For example, one of the best-studied bacteria for the production of ALG is *Pseudomonas aeruginosa* (*P. aeruginosa*). This Gram-negative bacterium is an opportunistic pathogen. Although a non-pathogenic strain of *P. aeruginosa* was successfully engineered [107], more studies are required to produce ALG with specific physicochemical properties. Furthermore, studies on optimizing the fermentation process are necessary before large-scale ALG production for commercialization can be carried out.

Finally, the limited resources of EPSs are the result of the small quantity of EPSs that can be isolated during extractions. An effective method should be developed for obtaining EPSs, especially for their synthesis, which could result in a greater EPS supply. Modification of EPS structures or chemical synthesis of EPSs could also be of great importance for developing specific side chains or changing single monosaccharides, as well as illuminating or boosting specific functions of EPSs [35].

## Figures and Tables

**Table 1:** Example of research on the biomedical applications of bacterial exopolysaccharides (EPSs).

Type of EPS	Source of EPS	Researched Biomedical Application	Reference
Cellulose	*Gluconacetobacter xylinum*	A bacterial cellulose–vaccarin wound dressing	[13]
	*Acetobacter xylinum, Pseudomonas* sp., *Agrobacterium Rhizobium* sp.	Wound healing and tissue-engineered blood vessels	[10]
	*Ochrobactrum pseudintermedium*	Surfactant for antibacterial properties	[14]
Dextran	*Leuconostoc* spp.	Nanogels for intracellular drug delivery	[15]
		Drug encapsulating agent	[16]
	*Leuconostoc mesenteroides* strain 7E	Synthetic blood volume expander, drug encapsulating agent, dextran–hemaglobin conjugate as a blood substitute	[10,17]
	*Weissella confuse*	Anti-biofilm activity against *Candida albicans* SC5314 strain	[18]
		Scaffold for L-926 fibroblasts	[19]
		Dextran–chitosan in situ gelling for adhesive properties	[20]
	*Lactobacillus reuteri*, *Lactobacillus sakei*, *Lactobacillus fermentum*, *Lactobacillus parabuchneri*	Plasma substitute	[9]
Xanthan gum	*Xanthomonas campestris*	Hydrogels for an ionic strength-sensitive gentamicin release system	[21]
		Carriers for drugs and proteins, as scaffolds for cells, tablets, or hydrogels for drug delivery	[22]
		Intra-articular injections for the treatment of osteoarthritis, adjuvant in the immune system, hydrogel for antitumor properties and wound dressing	[23]
Hyaluronic acid	*Bifidobacterium*	Nanoparticles for the treatment of diabetes in rats in vivo	[24]
		Anticancer drug delivery to SCC7 carcinoma cell line, human breast adenocarcinoma (MCF-7) cell line, and human lung epithelial adenocarcinoma (A549)	[16]
	*Streptococcus* sp.	Ophthalmic surgery, wound healing	[10]
	*Streptococcus equi, Streptococcus zooepidemicus*	Tissue engineering and bone regeneration, drug delivery, cancer diagnosis (CD44 interaction)	[25]
	*Cyanobacteria*	Wound healing	[26]
		Composite scaffold for skin regeneration	[27]
Alginate		Wound dressing containing *Alhagi maurorum* extract	[28]
		Wound dressing, scaffold for drug delivery	[29]
		Hydrogels for wound dressing	[23]
	*Pseudomonas aeruginosa*	Alginate–chitosan hydrogels for human cell encapsulation	[30]
	*Azotobacter vinelandii*	Alginate-based hydrogel for inducing retinal pigment epithelium regeneration	[31]
	*Nostoc* sp.	Aerogel for drug delivery and tissue engineering	[25]
	*Pseudomonas*, *Azotobacter*	Gel for drug and protein delivery, wound dressing, cell culture, tissue regeneration, bone regeneration, islet transplantation for treatment of type 1 diabetes, chondrocyte transplantation for treatment of urinary incontinence and vesicoureteral reflux	[32]
Kefiran	Kefir grains purchased from a household in Guimarães, Portugal	Cryogel scaffold for the delivery of diclofenac	[33]
	Kefir grains obtained from a household in Golestan, Iran	Nanofiber loaded with doxycycline for wound dressing	[34]
	*Lactobacillus hilgardii*, *L. rhamnosus*, *L. kefir*, *L. kefiranofascien*	Antitumor properties	[10]
	*L. kefiranofascien*	Mucosal adjuvant	[35]
Gellan	*Paenibacillus polymyxa*	Acetylated gellan for antioxidant and antitumor properties	[36]
	*Pseudomonas elodea*	Gellan film as an implant for insulin delivery	[37]
	*Pseudomonas elodea*, *Sphingomonas paucimobilis*	Administration of nasal formulations	[4]
	*Sphingomonas paucimobilis*	Tissue engineering and bone regeneration	[25]
	n.a.	Injectable gellan-based hydrogel to treat a bone defect	[38,39]
		Nano-hydrogel for drug delivery	[19]
		Composite scaffold for skin regeneration	[27]
	Lactic acid bacteria	Microencapsulation matrix for drug delivery	[12]
Levan	*Halomonas smyrnensis*	Levan-based scaffold for tissue engineering	[40]
		Multilayer film for the enhancement of live cell adhesive properties, harbor aldehyde groups for antitumor properties	[41]
		Disintegration agent in immediate-release tablets	[16]
		Sulfated levan for cardiac tissue engineering	[42]
	*Bacillus subtilis, Streptococcus mutans, Streptococcus salivarius*	Hypocholesterolemic agent, adhesive, antitumor properties	[9]
	*Gluconacetobacter xylinus*, *Rah nella aquatilis*, *Zymomonas mobilis*, *Microbacterium laevaniformans*	G-levan, R-levan, Z-levan, and M-levan for antitumor properties	[43]
	*Zymomonas mobilis*	Nanoparticles for wound healing, encapsulation agent	[25]
Curdlan	*Alcaligenes faecalis*, *Rhizobium radiobacter*, *Agrobacterium* sp.	Nanofibers as carriers of tetracycline hydrochloride	[44]
		Curdlan-based nanoparticles for entrapping antitumor drug and epirubicin	[45]
		Nanoparticles in bimolecular imaging, molecular diagnostics, drug delivery for cancer therapeutics and bioengineering, hydrogels for protein delivery and wound healing	[10]
		Anti-tuberculous activity	[46]
	*Paenibacillus polymyxa*	Curdlan sulfate as a vaccine against the infection of hepatitis B virus (HBV), treatment of severe malaria	[47]
		Antioxidant and antitumor activities	[48]

## Data Availability

Not applicable.
